# Radiomics analysis of dual-energy CT-derived iodine maps for predicting histopathologic grading of pancreatic ductal adenocarcinoma: a two-center study

**DOI:** 10.3389/fmed.2026.1769626

**Published:** 2026-03-17

**Authors:** Xinwei Wang, Dan Zeng, Zuhua Song, Jie Huang, Qian Liu, Dan Zhang, Zhuoyue Tang

**Affiliations:** Department of Radiology, Chongqing General Hospital, Chongqing University, Chongqing, China

**Keywords:** dual-energy computed tomography, histopathologic grading, iodine map, pancreatic ductal adenocarcinoma, radiomics

## Abstract

**Objective:**

This study aimed to develop and validate a nomogram with radiomics features extracted from dual-energy computed tomography (DECT)-derived iodine maps for preoperatively predicting the histopathologic grading in pancreatic ductal adenocarcinoma (PDAC).

**Materials and methods:**

In this two-center retrospective analysis, 151 patients were enrolled (82 in the training set; 36 in the testing set, and 33 in the external validation set), all of whom underwent DECT imaging. The radiomics signature was developed using features extracted from DECT-derived portal venous phase (PVP) iodine maps. A clinical model was subsequently established based on significant clinical factors identified through multivariate analysis. The radiomics signature combined with clinically significant features was used to construct the final predictive model. Model performance was assessed through the area under the receiver operating characteristic curve (AUC) and decision curve analysis (DCA). The most predictive model was employed to construct the nomogram, with its calibration accuracy being assessed through calibration plot analysis.

**Results:**

The radiomics-clinical model, combining the radiomics signature, body mass index, and carbohydrate antigen 125 levels, showed strong predictive performance for predicting histopathologic grade in PDAC across the training, testing, and external validation datasets, with respective AUCs of 0.873, 0.836, and 0.862. The DCA and calibration curve demonstrated an enhanced overall benefit and demonstrated reliable consistency.

**Conclusion:**

The radiomics–clinical model exhibited strong performance in preoperatively predicting the histopathologic grading in patients with PDAC.

## Introduction

Pancreatic ductal adenocarcinoma (PDAC) constitutes the predominant histological subtype of pancreatic malignancy, characterized by an exceptionally poor prognosis evidenced by a five-year survival rate below 10% (1). Histopathologic grading is a significant independent prognostic factor for PDAC, the overall survival has been lower for patients with poorly differentiated tumors than for those with moderately differentiated and well-differentiated tumors (the median overall survival was 9.8 months vs. 18.0 months) (2, 3). Studies have revealed that well-differentiated and moderately differentiated PDAC patients can achieve long-term survival prognosis after receiving conventional surgery, while survival prognosis for the poorly differentiated PDAC patients receiving neoadjuvant therapy (NAT) is better than undergoing conventional surgery (4, 5). Therefore, accurate preoperative assessment of the tumor grade is essential to guide therapeutic strategies and improve patient outcomes.

Currently, the histopathological grading of pancreatic ductal adenocarcinoma (PDAC) prior to surgical intervention primarily depends on image-guided biopsy techniques, including endoscopic ultrasound-guided fine needle biopsy (EUS-FNB) and percutaneous biopsy assisted by either ultrasound or computed tomography (CT) (6). However, they are invasive processes with a high risk of inducing complications such as pancreatitis (7). Meanwhile, the specimens obtained by these ways cannot accurately reflect the characteristics of the entire lesion due to the heterogeneity of PDAC (8, 9). Thus, a noninvasive, precise method is required to evaluate the histopathologic grading of PDAC.

Radiomics uses advanced image analysis techniques and machine learning algorithms to process high-throughput features extracted from digital medical images, which can effectively assess the staging, differential diagnosis and prognosis of various types of tumors, and has become an important complementary means of tumor imaging (10–13). Dual-energy computed tomography (DECT) is a widely used diagnostic imaging tool that can differentiate between tissues using the characteristic attenuation properties of various energy levels, including 40–140 keV energy levels, iodine maps, and effective atomic number, surpassing the diagnostic performance of conventional CT (14). The combination of DECT and radiomics offers significant potential to improve the diagnostic and predictive power of radiomics. DECT combined with radiomics has demonstrated excellent performance in predicting PDAC lymph node metastasis, gastric cancer classification, and invasiveness of lung adenocarcinoma (15–17). However, the use of DECT combined with radiomics to predict the histopathologic grading of PDAC has not yet been reported.

DECT-derived iodine maps can enhance the contrast between PDAC and normal pancreatic parenchyma and also reflect the histopathologic grading of PDAC (18, 19). Previous study has shown that radiomics enables extraction of extensive quantitative feature sets from iodine maps, thereby obtaining more information about the tumor (16). Consequently, this study aimed to assess the predictive performance of radiomics features derived from DECT iodine maps for the histopathologic grading of PDAC.

## Materials and methods

### Patients

This retrospective study received approval from the review board, with waiver of informed consent granted due to the study’s non-interventional design (No. KY S2024-069-01). Data were obtained from consecutive patients with pathologically confirmed PDAC at center 1 between July 2021 and July 2024 and at center 2 between July 2022 and June 2024. The inclusion criteria were as follows: (1) pathologically confirmed PDAC based on postoperative specimens; (2) patients underwent DECT-enhanced scans 2 weeks before surgery; and (3) clear histopathologic grading results. The exclusion criteria comprised the following conditions: (1) patients received radiotherapy, chemotherapy, or chemoradiotherapy before surgery; (2) poor image quality, unsuitable for tumor assessment; (3) incomplete clinical or DECT imaging data; and (4) coexisting other primary tumors.

**Figure 1:**
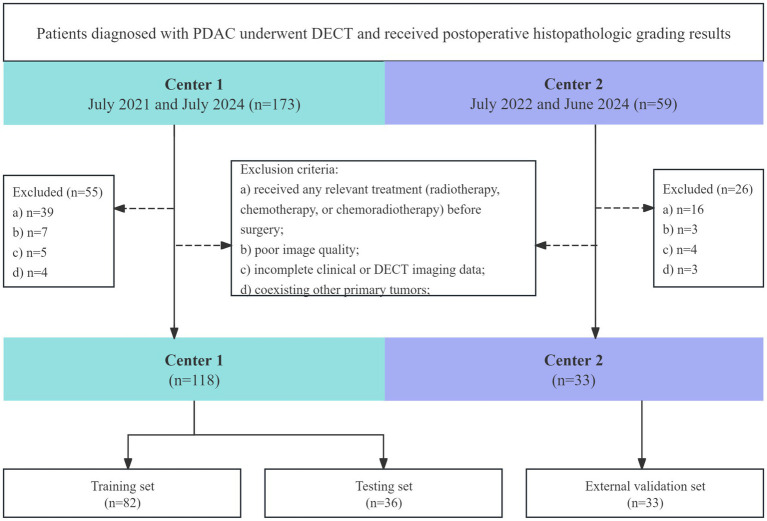
Flowchart of the study population. DECT, dual-layer spectral detector computed tomography; PDAC, pancreatic ductal adenocarcinoma.

Finally, participants included 151 patients (118 from center 1, 33 from center 2). Patients from center 1 were randomized in a 7:3 ratio into a training set (*n* = 82) and a testing set (*n* = 36). The flowchart of the patient selection is illustrated in Figure 1.

### Clinical features

Demographic and clinical parameters, including sex, age, and body mass index (BMI), were retrospectively collected from electronic medical records, albumin, total bilirubin, carbohydrate antigen 125 (CA125), CA19-9, lymphocyte-monocyte ratio (LMR), platelet-to-lymphocyte ratio (PLR), neutrophil-to-lymphocyte ratio (NLR), and platelet-to-white blood cell ratio (PWR).

### DECT image acquisition

All participants from both institutions underwent standardized DECT examinations according to protocol specifications detailed in Supplementary material 1.

### Histopathologic analysis

According to the 2019 World Health Organization (WHO) criteria, PDAC is classified into well-differentiated, moderately differentiated, and poorly differentiated categories. The histopathologic grading of tumors was determined from postoperative gross specimens. Consistent with established histopathological classifications, tumors were stratified into two groups: low-grade (LG; comprising moderately and well-differentiated tumors) and high-grade (HG; consisting of poorly differentiated tumors) (7, 20).

### Volumes of interest segmentation

**Figure 2:**
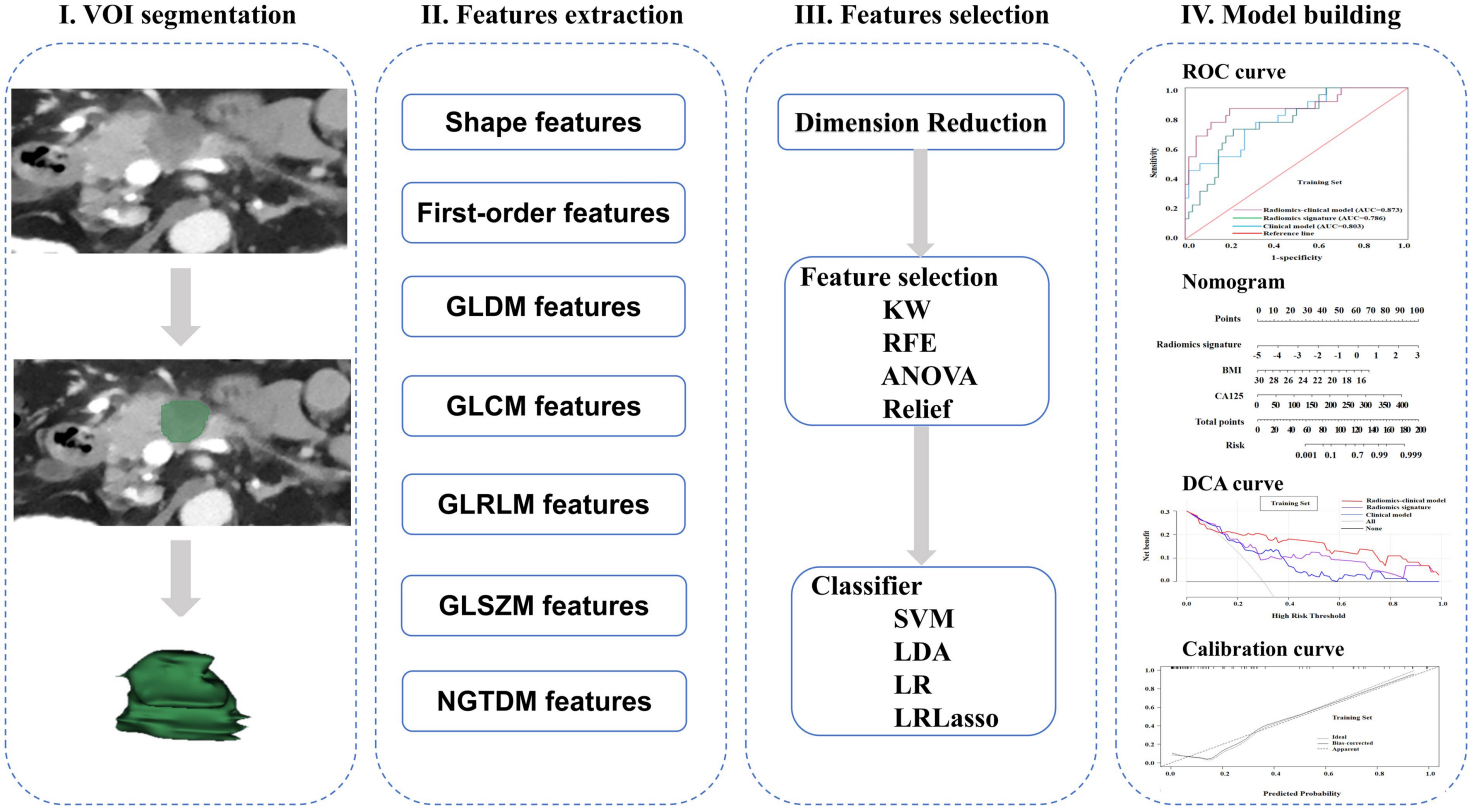
A schematic diagram for the whole radiomics and machine learning pipeline.

Figure 2 schematically represents the complete radiomics processing pipeline. Initially, Radiologist A manually outlined tumor boundaries on axial portal venous phase (PVP) CT images using 3D Slicer (open-source software, v5.6.2) to define the regions of interest. To evaluate intra-observer reliability, the same radiologist re-segmented 30 randomly selected cases after a two-week washout period. Additionally, Radiologist B independently annotated the same 30 cases to assess inter-observer agreement. Both radiologists performed segmentations without access to histopathological findings. Only radiomic features demonstrating intraclass correlation coefficients (ICCs) exceeding 0.75 in both intra- and inter-observer analyses were retained for subsequent evaluation.

### Radiomics extraction and analysis

The radiomic features were quantitatively characterized from DECT-derived PVP iodine maps using the open-source FeAture Explorer software (version 0.5.16) implemented in Python (3.7.6) (21). Prior to feature quantification, all CT datasets were spatially normalized to isotropic 1 mm^3^ voxels with intensity values standardized to a scale factor of 1,000. Subsequent image transformations incorporated multiple computational filters including quadratic, square root, gradient, logarithmic, wavelet, Laplacian of Gaussian, exponential, and three-dimensional local binary pattern operations. The comprehensive radiomic feature set encompassed first-order statistics, three-dimensional morphological descriptors, gray-level run length matrix parameters, gray-level size zone matrix characteristics, neighboring gray-tone difference matrix components, gray-level co-occurrence matrix indices, and gray-level dependence matrix metrics. Only radiomic features demonstrating good reproducibility (intra- and inter-observer ICC > 0.75) were retained for subsequent analysis. These retained features were then z-score normalized, followed by removal of redundant features with Pearson correlation coefficients > 0.99, before downstream feature selection and model construction. Multivariate feature selection was performed using analysis of variance, Kruskal-Wallis (KW) nonparametric testing, Relief-based feature weighting, and recursive feature elimination algorithms. Predictive modeling employed tenfold cross-validation across four machine learning classifiers: support vector machines with kernel optimization, linear discriminant analysis (LDA), conventional logistic regression, and LASSO-regularized logistic regression. Model performance was quantitatively assessed through receiver operating characteristic analysis, with the optimal radiomic signature selected based on maximal predictive accuracy in the independent test set.

Model discrimination was evaluated through receiver operating characteristic (ROC) analysis, with the radiomics signature demonstrating maximal predictive accuracy in the independent test set being designated as optimal.

### Development of clinical and radiomics–clinical model

The initial stage involved characterizing clinical parameters through univariate statistical evaluation. Subsequently, variables showing statistically significant associations (*p* < 0.05) in univariate analysis were included in the multivariable logistic regression model to establish clinical prediction models within the training set.

An integrated radiomics-clinical nomogram was constructed by merging the radiomic profile obtained from DECT-derived PVP iodine maps with clinically significant parameters using multivariate logistic regression. Multivariable analysis yielded odds ratios (OR) with 95% confidence intervals (CI) for all significant independent variables.

### Performance evaluation of the three models

The performances of the three models in all the sets using AUC with 95% CIs, sensitivity, and specificity, and decision curve analysis (DCA) for clinical net benefit quantification, while calibration curves assessed the nomogram’s prediction accuracy.

### Statistical analysis

Data normality was evaluated using the Shapiro–Wilk test, with normally distributed continuous variables expressed as mean ± standard deviation (SD), non-normal data as median (interquartile range), categorical variables analyzed by chi-square test, and continuous variables compared via Mann–Whitney U test or independent *t*-test as appropriate, adopting a two-tailed *p* < 0.05 threshold for significance, with all analyses conducted using R,^1^ SPSS (v26.0, IBM), and MedCalc (v18.2.1, MedCalc Software).

## Results

### Patient characteristics

The training set comprised 73.2% (60/82) of patients with LG tumors and 26.8% (22/82) of patients with HG tumors, and the testing set comprised 69.4% (25/36) of patients with LG tumors and 30.6% (11/36) of patients with HG tumors. The external validation set comprised 60.1% (13/33) of patients with LG tumors and 39.9% (20/33) of patients with HG tumors. Table 1 summarized the clinical characteristics of PDAC in training, testing, and external validation sets, and showed significant differences in BMI and ALB in three groups.

**Table 1:** Clinical features of PDAC patients.

Features	Training set (*n* = 82)	Testing set (*n* = 36)	External validation set (*n* = 33)	*p*
Age, y	60 (54.75,69.25)	62 (55,69.75)	61 (56,69)	0.898
BMI	22.04 (20.61,24.78)	21.87 (20.80,24.72)	20.13 (19.21,21.31)	0.001
Gender, *n* (%)				0.175
Male	51 (62.2)	16 (44.4)	18 (54.5)	
Female	31 (37.8)	20 (55.6)	15 (45.5)	
CA19-9	407.24 ± 610.22	258.64 ± 477.18	247.43 ± 282.44	0.201
CEA	4.96 ± 10.25	8.45 ± 30.51	15.75 ± 64.78	0.317
CA125	36.53 ± 65.53	104.40 ± 331.04	88.99 ± 139.90	0.117
ALB	39.85 (36.76,42.63)	37.80 (35.93,39.88)	41.40 (38.30,44.50)	0.002
TBil	66.78 ± 95.04	94.93 ± 104.74	115.83 ± 129.07	0.065
PLR	211.19 ± 105.72	226.28 ± 128.69	239.00 ± 150.29	0.520
NLR	4.09 ± 4.03	4.47 ± 3.02	5.42 ± 9.61	0.511
LMR	2.99 (2.16,4.01)	3.02 (2.04,3.89)	2.02 (1.40,3.70)	0.061
PWR	40.38 (29.62,51.16)	35.88 (28.39,48.48)	37.59 (30.67,54.22)	0.478

PDAC, pancreatic ductal adenocarcinoma; BMI, body mass index, ALB, albumen; TBil, total bilirubin; CA19-9, carbohydrate antigen 19–9; CA125, carbohydrate antigen 125; CEA, carcinoembryonic antigen; PLR, platelet/lymphocyte; NLR, neutrophil/lymphocyte; LMR, lymphocyte/monocyte; WBC, platelet/white blood cell.

### Clinical model development

Univariate analysis of the training set revealed significant differences between HG and LG groups in BMI, sex, CA125 levels, NLR, and LMR (Table 2). Subsequent multivariate logistic regression analysis identified BMI (OR = 0.723) and CA125 (OR = 1.018) as independent predictors of PDAC histopathologic grading (Table 3). Based on these findings, we developed a clinical prediction model incorporating these two significant variables.

**Table 2:** Univariate analysis to differentiate between HG and LG groups in the training set.

Variables	HG (*n* = 22)	LG (*n* = 60)	F/Z/c²	*p*
Age, y	59.5 (54.25,68)	60 (54.25,70.75)	-0.175	0.533
BMI	20.81 (19.74,22.49)	23.26 (20.81,25.28)	-1.131	0.005
Gender, *n* (%)			1.283	0.006
Male	16 (72.7)	35 (58.3)		
Female	6 (27.3)	25 (41.7)		
CA19-9, *n* (%)	602.20±789.86	335.75±519.41	4.407	0.080
CEA, *n* (%)	6.33±12.38	4.46±9.42	2.840	0.466
CA125, *n* (%)	81.61±112.25	19.99±19.38	10.275	<0.001
ALB	39.70 (36.77,41.97)	39.85 (36.72,42.80)	-2.063	0.467
TBil	70.57±105.98	65.39±91.62	1.260	0.828
PLR	239.24±131.51	200.91±93.74	-0.247	0.147
NLR	5.91±6.90	3.42±1.90	-0.007	0.012
LMR	2.60 (1.51,3.39)	3.15 (2.36,4.16)	-0.245	0.041
PWR	27.29 (36.66,51.17)	40.57 (32.60,51.33)	-1.093	0.426
Radiomics signature	0.32 (-0.20,1.19)	-0.60 (-1.47,0.22)	-0.368	<0.001

HG, high-grade; LG, low-grade; BMI, body mass index, ALB albumen, TBil total bilirubin; CA19-9, carbohydrate antigen 19–9; CA125, carbohydrate antigen 125; CEA, carcinoembryonic antigen; PLR, platelet/lymphocyte; NLR, neutrophil/lymphocyte; LMR, lymphocyte/monocyte; WBC, platelet/white blood cell.

**Table 3:** Multivariate logistic regression analysis in the training set.

Variables	Odds ratio	95% CI	*p*
Radiomics signature	2.668	1.379-5.160	0.004
BMI	0.723	0.555-0.942	0.016
CA125	1.018	1.002-1.033	0.023
Gender	1.706	0.454-6.406	0.492
NLR	1.165	0.825-1.6476	0.386
LMR	0.824	0.446-1.524	0.538

CI, confidence interval; CA125, carbohydrate antigen 125; BMI, body mass index, NLR, neutrophil/lymphocyte, LMR, lymphocyte/monocyte.

### Radiomics signature and radiomics–clinical model development

**Figure 3:**
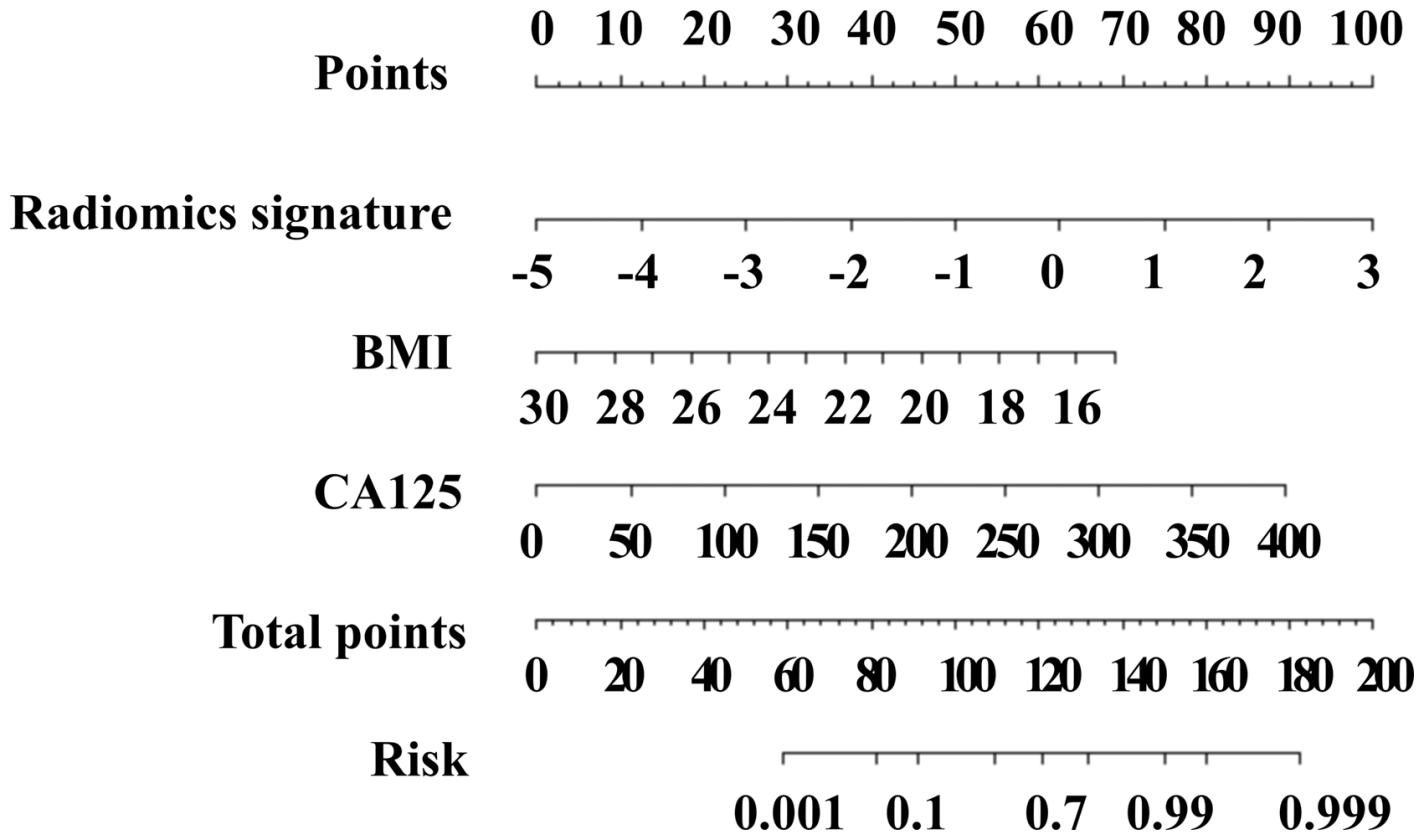
The radiomics-clinical nomogram incorporating the radiomics signature, body mass index (BMI), and carbohydrate antigen 125 (CA125). The method for calculating the risk of poorly differentiated was as follows. First, points for each predictor are assigned by corresponding values from the “Points” axis. Second, the “Total points” is computed by summing up all predictors’ points. Third, a vertical line should be drawn down the total points to get the risk of poorly differentiated.

Dimensionality reduction using Pearson correlation coefficient (PCC) analysis, combined with the KW test for feature selection and LDA for classification, yielded an optimal set of 14 discriminative features. The mathematical formulation of the final radiomics signature is provided in Supplementary material 2. The diagnostic performance of each classifier is presented in Supplementary material 3. Multivariate logistic regression analysis of the training set identified three independent predictors of PDAC histopathological grading, the results are in the Table 3. These variables were integrated to construct a combined radiomics-clinical prediction model, which was subsequently visualized as a clinically applicable nomogram (Figure 3).

### Evaluation and comparison of model performance

**Figure 4:**
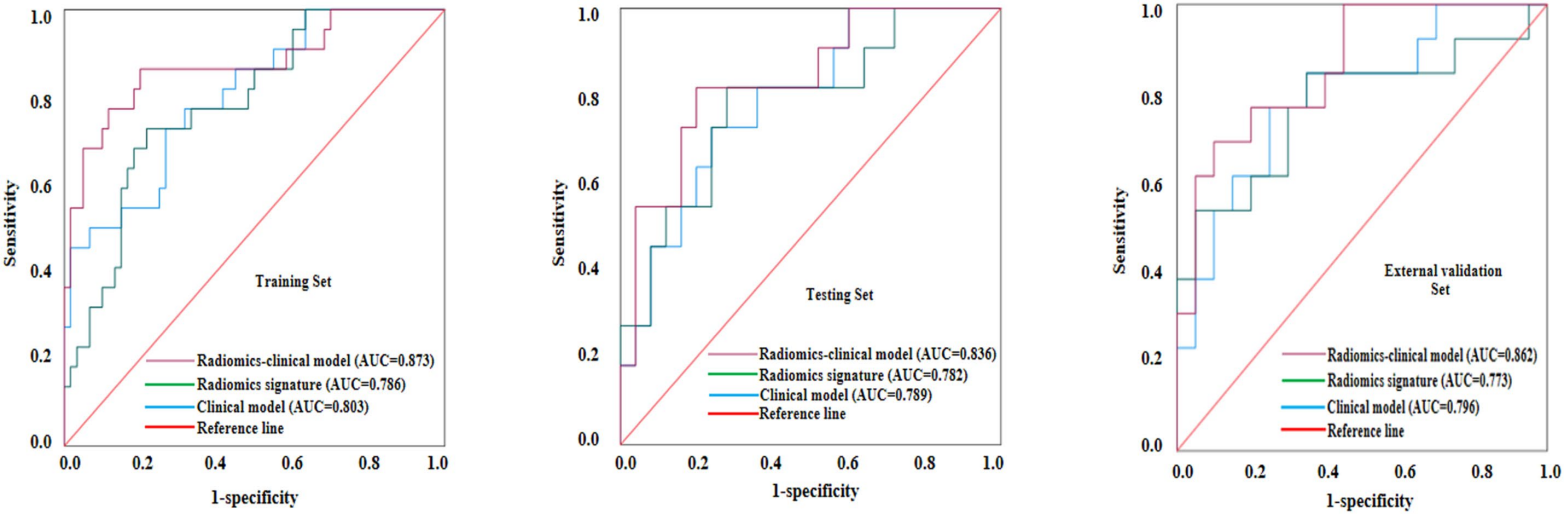
Receiver operating characteristic (ROC) curves of the clinical model, radiomics signature, and radiomics-clinical nomogram for predicting histopathologic grading for patients with pancreatic ductal adenocarcinoma (PDAC) in the training, testing, and external validation sets.

**Figure 5:**
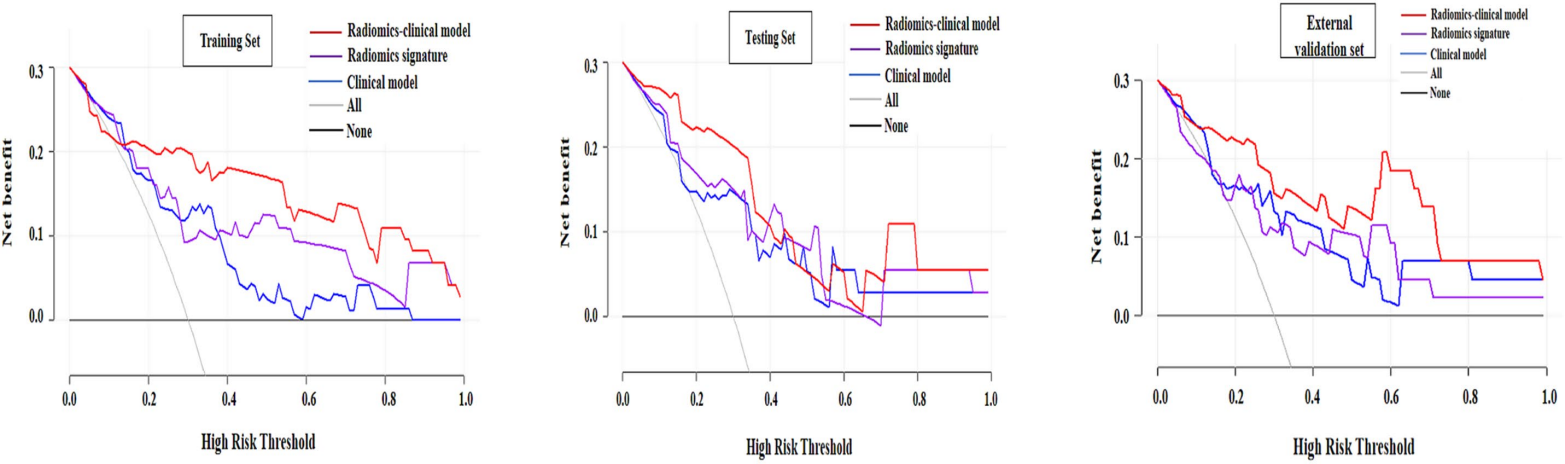
Decision curve analysis (DCA) for clinical model, radiomics signature, and radiomics-clinical nomogram in the training, testing, and external validation sets. The *x*-axis indicates threshold probability and the *y*-axis indicates the net benefit. Using the radiomics-clinical nomogram can obtain higher net benefit than treat-all strategy and treat-none strategy.

**Figure 6:**
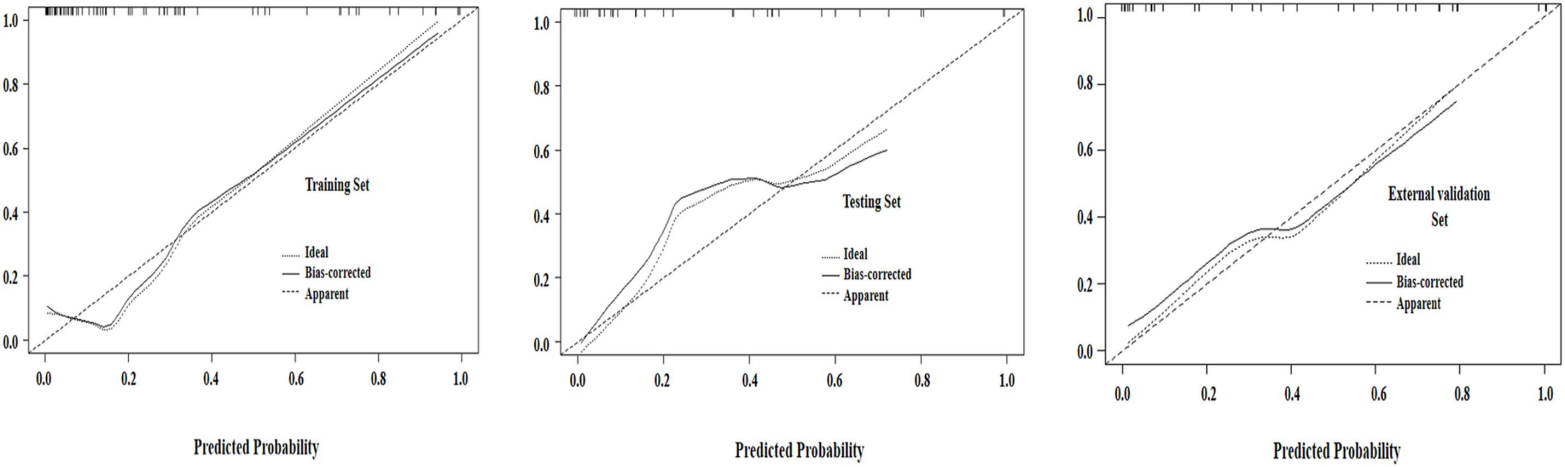
Calibration curves for the radiomics-clinical nomogram in the training, testing, and external validation sets. The 45° straight line represents an ideal evaluation. The solid line indicates the correction of bias of the nomogram. A closer distance between two lines indicates better goodness-of-fit.

Table 4 presents the AUC, sensitivity, specificity of the 3 models in all sets. The ROC analysis of the 3 models for the histopathologic grading of PDAC in all sets was illustrated in Figure 4. The clinical model demonstrated superior diagnostic efficacy in predicting the histopathologic grading of patients with PDAC, with an AUC of 0.803 (95% CI: 0.688–0.901) in the training set, 0.789 (95% CI: 0.654–0.928) in the testing set and 0.796 (95% CI: 0.639–0.954) in the external validation set. Both the radiomics signature and the radiomics–clinical model exhibited superior diagnostic efficacy in predicting the histopathologic grading of patients with PDAC, with AUCs of 0.786 (95% CI: 0.677–0.893), 0.873 (95% CI: 0.766–0.968) and in the training set and 0.782 (95% CI: 0.611–0.953), 0.836 (95% CI: 0.758–0.962) in the testing set, and 0.773 (95% CI: 0.584–0.952), 0.862 (95% CI: 0.736–0.987) in the external validation set, respectively. The DeLong tests revealed no significant performance differences between radiomics-clinical models and clinical models, nor between radiomics models and clinical models, across the training (*p* = 0.071 and *p* = 0.385), test (*p* = 0.356 and *p* = 0.353), and external validation cohorts (*p* = 0.072 and *p* = 0.360). However, the radiomics-clinical model demonstrated significantly higher AUC values than the radiomics model in the training (*p* = 0.034), testing (*p* = 0.042), and external validation cohorts (*p* = 0.038). The DCA demonstrated superior net benefit for the radiomics-clinical model across clinically relevant threshold probabilities compared to either model alone, confirming its enhanced clinical utility (Figure 5). In all the sets, the radiomics-clinical model exhibited strong calibration, with predicted probabilities closely matching actual observed frequencies (Figure 6).

**Table 4:** Predictive performance of the clinical model, radiomics signature, and radiomics-clinical models.

Models	Set	AUC (95% CI)	SEN	SPE
Clinical model	Training	0.803 (0.724-0.886)	0.727	0.733
	Testing	0.789 (0.574-0.866)	0.727	0.760
	External validation	0.796 (0.639-0.954)	0.769	0.750
Radiomics signature	Training	0.786 (0.926-0.988)	0.636	0.760
	Testing	0.782 (0.817-0.985)	0.636	0.760
	External validation	0.773 (0.594-0.952)	0.846	0.650
Radiomics-clinical model	Training	0.873 (0.767-0.962)	0.864	0.800
	Testing	0.836 (0.732-0.957)	0.818	0.800
	External validation	0.862 (0.736-0.987)	0.792	0.800

AUC, area under the receiver operating characteristic curve; CI, confidence interval; SEN, sensitivity; SPE specificity.

## Discussion

This retrospective study established a noninvasive and effective radiomics–clinical model integrating the radiomics signature based on DECT-derived PVP iodine maps, BMI, and CA125 for preoperative prediction of the histologic grading of patients with PDAC. The nomogram exhibited favorable predictive performance and calibration.

Both univariate and multivariate logistic regression analyses identified BMI and CA125 as statistically independent predictors of the histologic grading of patients with PDAC. CA125 is a biomarker associated with the prognosis of patients with PDAC; a high preoperative CA125 level is an independent predictor of lower overall and disease-free survival in patients with PDAC and indicates a higher probability of occult metastases than in those with low CA125 levels (22, 23). HG tumors tend to have stronger tumor angiogenesis and higher cellularity than LG tumors, indicating a poorer prognosis (24). Importantly, BMI should be interpreted cautiously in PDAC. In our multivariable model (HG = 1), BMI showed an OR < 1, suggesting that lower BMI was independently associated with high-grade tumors. This finding may reflect cancer-related weight loss and nutritional decline in more aggressive disease; however, BMI does not distinguish adiposity from skeletal muscle loss, and unmeasured body-composition changes (e.g., sarcopenia) may confound this association (25, 26). Therefore, while CA125 consistently supported a more aggressive phenotype, the biological interpretation of BMI warrants further investigation in future studies incorporating CT-derived body-composition indices.

Radiomics extracts high-throughput quantitative imaging features and responds to tumor heterogeneity (27). Recent studies have successfully used DECT in combination with radiomics to predict PDAC lymph node metastasis, gastric cancer classification, and invasiveness of lung adenocarcinoma (15–17). DECT-derived iodine maps provide quantitative information on tissue perfusion and iodine uptake (e.g., iodine concentration), and prior studies have reported associations between iodine-based metrics and tumor aggressiveness (18, 19). However, conventional evaluation typically relies on a limited number of summary measures—often the mean iodine concentration within a small ROI—thereby “averaging out” intratumoral spatial heterogeneity into a single value. In contrast, iodine-map radiomics characterizes the whole-lesion spatial distribution of iodine uptake by extracting higher-order features (e.g., texture and multi-scale heterogeneity descriptors) that are not readily appreciable by visual inspection or captured by a single average metric. This enables microscopic heterogeneity in iodine distribution to be quantified as reproducible mathematical features, providing incremental information beyond iodine concentration alone (28, 29). In this study, the radiomics signature constructed based on the PVP iodine maps demonstrated favorable efficacy in all the training (AUC = 0.786), testing (AUC = 0.782) and external validation (AUC = 0.773) sets. Ultimately, we selected 14 radiomics features that were associated with the histologic grading of PDAC, with texture-derived metrics accounting for a substantially larger proportion than first-order features. Texture features quantify spatial relationships and dispersion patterns of voxel intensities and are commonly interpreted as imaging surrogates of intratumoral heterogeneity and structural complexity (30). From a histopathologic perspective, higher-grade PDAC is frequently accompanied by increased cellularity and architectural disorganization, heterogeneous desmoplastic stromal reaction, and occasional microscopic necrosis, these microstructural alterations may lead to spatially heterogeneous perfusion and iodine distribution, thereby contributing to a more heterogeneous textural appearance on iodine maps (31). Wavelet-based features further decompose images into multi-scale frequency components and may amplify subtle heterogeneity signals potentially related to such microstructural variations, consistent with prior radiomics studies exploring PDAC microenvironmental biomarkers (32–34). Notably, because no quantitative radiology–pathology correlation analysis was performed in this study, the above interpretations should be considered biologically plausible inferences and warrant validation in future radiology–pathology correlation studies. Machine learning algorithms combined with radiomics analysis has demonstrated potential in predicting the histologic grading of various tumors (35–38). Integrating the optimal radiomics signature with two clinically significant predictors (BMI and CA125), we developed and validated a radiomics-clinical nomogram that quantifies individualized risk scores. This nomogram demonstrated the highest AUC of 0.873, 0.836 and 0.862 in the training, testing and external validation sets, respectively, demonstrating improved predictive efficacy compared with radiomics signature alone. DCA indicated that patients can achieve great clinical net benefits from radiomics–clinical models. Besides, the calibration curve demonstrated favorable agreement between nomogram prediction and actual observed probability. These results suggest that the histologic grading of patients with PDAC can be evaluated noninvasively before surgery, providing an objective basis for further clinical treatment.

This study has several limitations that should be acknowledged. First, this was a retrospective study, so large-sample, multicenter, prospective studies are needed in the future to validate the robustness and generalizability of the model. Second, arterial/pancreatic-phase iodine maps were not incorporated. Because early-phase acquisitions may provide complementary perfusion-related information relevant to tumor grade, future multicenter prospective studies with harmonized acquisition timing are warranted to assess the added value of multiphasic iodine-map radiomics. Finally, this study focused only on evaluating the predictive accuracy of DECT for histologic grading in PDAC. Further analysis is needed to assess the prognosis of patients with PDAC.

## Conclusion

The radiomics–clinical model, established combining the radiomics signature of DECT-derived iodine maps with the clinical features, exhibited strong performance in preoperatively predicting the histopathologic grading of patients with PDAC. This approach may help clinicians in personalize treatment.

## Data Availability

The original contributions presented in the study are included in the article/Supplementary material, further inquiries can be directed to the corresponding author.

## Footnotes

1 http://www.R-project.org.
