# Supplementary material for: Radiomics analysis of dual-energy CT-derived iodine maps for predicting histopathologic grading of pancreatic ductal adenocarcinoma: a two-center study
Source: Front Med (Lausanne). 2026 Mar 17;13:1769626. doi: 10.3389/fmed.2026.1769626 (PMC13035504; doi:10.3389/fmed.2026.1769626)
Supplement: Supplementary file 1 [file Supplementary_file_1.docx]

**Supplement Materials 1**

**Dual-energy computed tomography (DECT) image acquisition**

In center 1, the DECT images were acquired on a spectral CT scanner (IQon Spectral CT, Philips Healthcare). Typical imaging parameters were as follows: tube voltage, 120 kV; smart mAs; rotation time, 0.5 s; detector collimation, 64 × 0.625 mm; matrix, 512 × 512; reconstruction thickness, 1 mm. A nonionic contrast medium (Ioversol, 350 mg/ml) was injected with an automatic injector at a dose of 1.5 ml/kg at 2.5-3.5 ml/s, followed by 30 ml of a saline flush at the same rate. For image acquisition, an automatic bolus tracking technique was used. Arterial phase scan started with a delay of 12s after passing the predetermined threshold of 150 HU within the abdominal aorta ; Portal vein phase scan started 35s after the arterial phase.

In center 2, the DECT images were acquired on a spectral CT scanner (FORCE, Siemens Healthineers). Typical imaging parameters were as follows: tube voltage, A tube 100 kV and B tube Sn 150 kV; automatic modulation of tube current (reference values: A tube 280 mAs, B tube 140 mAs); rotation time, 0.5 s; detector collimation, 128 × 0.6 mm; matrix, 512 × 512; reconstruction thickness, 1.5 mm with an interval of 1.0 mm. A nonionic contrast medium (Omnipaque, GE Healthcare) was injected at a dose of 1-1.5 ml/kg at a rate of 3-4 ml/s, followed by 30 ml of a saline flush. For image acquisition, an automatic bolus tracking technique was used, with the arterial phase scan starting 9 s after reaching a threshold of 180 HU in the target vessel. The portal vein phase scan started 30 s after the arterial phase.

**Supplement Materials 2**

**Radscore=** Iodine_exponential_firstorder_TotalEnergy × 0.470 +

Iodine_lbp-3D-m2_glrlm_RunEntropy × 0.191 - Iodine_log-sigma-1-mm-3D_gldm_LargeDependenceHighGrayLevelEmphasis ×

0.077 + Iodine_log-sigma-1-mm-3D_glrlm_LongRunHighGrayLevelEmphasis ×

0.211 - Iodine_square_firstorder_TotalEnergy ×0.752 +

Iodine_square_gldm_SmallDependenceLowGrayLevelEmphasis × 0.448 -

Iodine_wavelet-HHH_glcm_ClusterShade 0.197 -

Iodine_wavelet-HLH_glcm_Correlation 0.828 -

Iodine_wavelet-HLH_gldm_DependenceNonUniformityNormalized ×

0.311 - Iodine_wavelet-HLH_ngtdm_Complexity 0.549 -

Iodine_wavelet-HLL_glszm_SmallAreaEmphasis - 0.642 -

Iodine_wavelet-LLH_gldm_DependenceNonUniformityNormalized 0.446 +

Iodine_wavelet-LLL_glszm_LargeAreaHighGrayLevelEmphasis × 0.359 Iodine_wavelet-LLL_ngtdm_Complexity × 1.145

**Supplement Materials 3**

| The diagnostic performance of of Four Machine Learning Classifiers | | |
| --- | --- | --- |
| Model | Set | AUC |
| Zscore_PCC_KW_14_LDA | training | 0.786 |
|  | testing | 0.782 |
|  | external validation | 0.773 |
| Zscore_PCC_KW_7_LRLasso | training | 0.734 |
|  | testing | 0.710 |
|  | external validation | 0.721 |
| Zscore_PCC_KW_6_LR | training | 0.767 |
|  | testing | 0.741 |
|  | external validation | 0.728 |
| Zscore_PCC_KW_8_SVM | training | 0.702 |
|  | testing | 0.726 |
|  | external validation | 0.711 |
| Zscore_PCC_ANOVA_11_LR | training | 0.672 |
|  | testing | 0.691 |
|  | external validation | 0.660 |
| Zscore_PCC_ANOVA_9_LRLasso | training | 0.667 |
|  | testing | 0.627 |
|  | external validation | 0.662 |
| Zscore_PCC_ANOVA_6_LDA | training | 0.645 |
|  | testing | 0.664 |
|  | external validation | 0.620 |
| Zscore_PCC_ANOVA_5_SVM | training | 0.671 |
|  | testing | 0.629 |
|  | external validation | 0.634 |
| Zscore_PCC_RFE_5_LDA | training | 0.723 |
|  | testing | 0.700 |
|  | external validation | 0.667 |
| Zscore_PCC_RFE_3_LRLasso | training | 0.689 |
|  | testing | 0.567 |
|  | external validation | 0.582 |
| Zscore_PCC_RFE_4_SVM | training | 0.714 |
|  | testing | 0.613 |
|  | external validation | 0.678 |
| Zscore_PCC_RFE_6_LR | training | 0.729 |
|  | testing | 0.604 |
|  | external validation | 0.618 |
| Zscore_PCC_Relief_10_LDA | training | 0.670 |
|  | testing | 0.554 |
|  | external validation | 0.572 |
| Zscore_PCC_Relief_7_SVM | training | 0.657 |
|  | testing | 0.638 |
|  | external validation | 0.644 |
| Zscore_PCC_Relief_9_LR | training | 0.667 |
|  | testing | 0.556 |
|  | external validation | 0.590 |
| Zscore_PCC_Relief_6_LRLasso | training | 0.653 |
|  | testing | 0.545 |
|  | external validation | 0.598 |
| ANOVA, analysis of variance; AUC, area under the curve; KW, kruskal-wallis; LDA, linear discriminant analysis; LR, logistic regression; LRLasso, logistic regression using Lasso; RFE, recursive feature elimination; SVM, support vector machine. | | |
